# African swine fever in wild boar: investigating model assumptions and structure

**DOI:** 10.1098/rsos.231319

**Published:** 2024-05-15

**Authors:** Callum Shaw, Angus McLure, Kathryn Glass

**Affiliations:** ^1^ National Centre for Epidemiology and Population Health, Australian National University, Canberra, Australian Capital Territory 2601, Australia

**Keywords:** African swine fever, mathematical modelling, infectious disease control, wild boar

## Abstract

African swine fever (ASF) is a highly virulent viral disease that affects domestic pigs and wild boar. Current ASF transmission in Europe is in part driven by wild boar populations, which act as a disease reservoir. Wild boar are abundant throughout Europe and are highly social animals with complex social organization. Despite the known importance of wild boar in ASF spread and persistence, knowledge gaps remain surrounding wild boar transmission. We developed a wild boar modelling framework to investigate the influence of contact-density functions and wild boar social structure on disease dynamics. The framework included an ordinary differential equation model, a homogeneous stochastic model and various network-based stochastic models that explicitly included wild boar social grouping. We found that power-law functions (transmission ∝ density^0.5^) and frequency-based contact-density functions were best able to reproduce recent Baltic outbreaks; however, power-law function models predicted considerable carcass transmission, while frequency-based models had negligible carcass transmission. Furthermore, increased model heterogeneity caused a decrease in the relative importance of carcass-based transmission. The transmission pathways predicted by each model type affected the efficacy of idealized interventions, which highlights the importance of evaluating model type and structure when modelling systems with significant uncertainties.

## 1. Introduction

African swine fever (ASF) is a viral haemorrhagic disease caused by the African swine fever virus (ASFV), which affects both domestic pigs and wild boar populations. There is currently no vaccine, and highly virulent strains of ASFV can result in close to 100% mortality [1], while infection from moderately virulent strains has lower mortality, ranging from 30% to 70% [2]. ASF outbreaks have the potential to devastate pig production. ASF outbreaks in China have led to the culling of 1.2 million pigs, and the outbreaks have had an estimated economic impact of 0.78% of China’s 2019 gross domestic product [3]. Similarly, outbreaks in wild boar in Europe have caused significant population decline [4]. Owing to this high potential for serious economic losses, ASF has been declared a notifiable disease by the World Organisation for Animal Health [5].

ASF was first identified in Kenya in 1921, after the introduction of European domestic pigs [6]. Genotype I ASFV was first reported in Europe in 1957 when it was discovered in Portugal. ASF was subsequently reported in Brazil and a number of Caribbean and European countries [7]. In the summer of 2007, there was an ASF outbreak in Georgia caused by the highly virulent genotype II ASFV. This genotype quickly spread through the Caucasus region and entered the Russian Federation by November 2007 [8]. Despite control efforts, the virus has since reached Eastern Europe and the European Union, where wild boar have been a key driver of the spread [9,10]. In mid-2018, genotype II ASFV reached northeastern China [11], and by early 2019, ASF had spread to 25 provinces in China [12]. Since its detection in China, there have been outbreaks in many East and Southeast Asian countries [13].

The epidemiology of ASF is complex, with multiple transmission pathways [14]. ASF can be spread through either direct or indirect contact; indirect transmission is possible as ASFV can remain viable in excretions of infected pigs for days [15] and viable in blood for months [16]. Furthermore, infectious ASFV has been found to last several days in various soil matrices [17] and in shipped feed [18]. Wild boar have been an important driver of the recent ASF outbreak in Eastern Europe [19] and South Korea [20]. Environmental transmission is a key component of transmission in wild boar populations; viable ASFV has been detected in wild boar carcasses for weeks after death [21], and wild boar have been observed interacting with conspecific carcasses and the environment around said carcasses [22]. The importance of infected carcasses in the continued persistence of ASF in Eastern European wild boar populations is supported by recent modelling work [23–26].

Wild boar are highly social animals and are often observed in groups [27]. Most often, groups are made up of one or more sows and their piglets, but can consist of young males or females [28]. Conversely, adult male boar are often solitary. This social structure introduces heterogeneity in wild boar contact patterns and has been found to influence ASF transmission dynamics [29]. However, wild boar population ecology is not homogeneous, as group size, home range area, density and growth rates vary between regions.

There have been numerous ASF modelling studies that have analysed transmission in wild pigs [30]. A majority of these studies have been stochastic individual-based models (IBM) (e.g. Halasa *et al*. [31], Pepin *et al.* [25], Gervasi & Guberti [26] and Han *et al.* [32]), but there have been several deterministic ordinary differential equation (ODE) models (e.g. O’Niell *et al.* [24] and Gervasi *et al.* [33]). The two model types implement social structure in different ways. The nature of the ODE models does not allow them to explicitly include the wild boar social structure, while the existing IBM include wild boar social groupings. The IBM run on a grid and each cell can contain groups of wild boar. In these models, ASF transmission occurs preferentially within family groups. Inter-group transmission generally occurs either from groups in directly neighbouring cells or from any group, with transmission strength decaying exponentially with distance. Furthermore, the models allow for pig dispersal, which can act as a route for disease transmission. While many modelling studies acknowledge the importance of wild boar social structure, there have been limited modelling studies where social structure and contact have been the focus. Both Pepin *et al.* [29] and Yang *et al.* [34] found that social structure affected wild boar disease dynamics and increased contact heterogeneity could improve transmission models. Contact heterogeneity has been shown to significantly affect disease dynamics [35,36]; however, despite the known importance of wild pig social dynamics in potential disease spread, they are poorly understood [37]. Therefore, alternative modelling approaches that investigate the relationship between population density and boar contact rates, and the impact of heterogeneous contact networks could gain insight into wild boar-mediated ASF transmission dynamics.

In this study, we develop a lightweight, highly adaptable ASF modelling framework that can model ASF dynamics in wild boar in a variety of settings. The framework ranges from simple ODE models to stochastic models that explicitly include various wild boar contact structures. The objective of the study is to highlight the impact of model assumptions, wild boar population structure characteristics and ASF transmission features on long-term ASF persistence in wild boar.

## 2. Model overview

**Figure 1.**
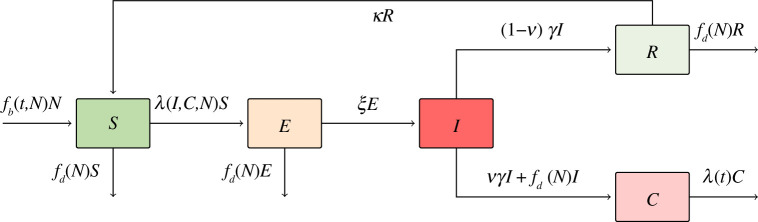
Underlying model structure, used in all tested models. There are five base classes: S , susceptible; E , exposed; I , infectious; R , recovered; and C , carcasses. N is the total living population, λ(I,C,N) is the force of infection, $f_{b}(t,N)$ is the recruitment rate, $f_{d}(N)$ is the death rate, λ(t) is the carcass decay rate, ζ is the latent rate, νγ is the ASF induced mortality rate, (1−ν)γ is the recovery rate, and κ is the waning immunity rate.

To accurately model an ASFV outbreak in naive wild boar populations, we extended the traditional compartmental susceptible (S) , infected (I) and recovered (R) models [38,39]. We included an exposed, non-infectious class (E) , and owing to the importance of potential carcass transmission, we included a class for the decaying carcasses of pigs that have recently died from ASF (C) . Piglets were excluded due to high first-year mortality, where a majority of deaths occur within the first three months [40]. Instead, only yearlings and adult pigs were included, which were combined to form a single adult class. The structure of the model is shown in figure 1, where N is the total living population, λ(I,C,N) is the force of infection, $f_{b}(t,N)$ is the time-dependent recruitment rate, $f_{d}(N)$ is the time-independent death rate, λ(t) is the carcass decay rate, ζ is the latent rate, νγ is the ASF induced mortality rate, (1−ν)γ is the recovery rate and κ is the waning immunity rate.

### 2.1. Recruitment

Seasonality in recruitment can have a large impact on disease dynamics in ecological systems [41]. Seasonality affects the timing of the recruitment of susceptible individuals, which can affect critical community size [42] and induce bifurcations and oscillations in disease incidence [43]. Furthermore, seasonality in wild boar births has been observed in a variety of geographic settings [44–47]. In our study, as piglets were not included, we modelled a temporally varying recruitment rate ( $f_{b}\,\left(t,N\right)$ ) that accounted for density effects. $f_{b}\,\left(t,N\right)$ was composed of a daily recruitment rate ( b⁢(t) ) that was scaled to account for population-level density effects. To include seasonality in recruitment, b⁢(t) was modelled with a periodic Gaussian function similar to that used by Peel *et al.* [42].


(2.1) $$b(t)=b_{0}\;\exp(-s\;\cos(\frac{\pi (t-\phi_{b})}{365}{)}^{2}),$$

where s alters pulse width, $\phi_{b}$ alters the phase of the pulse, and *b*_0_ is a scaling factor to ensure the correct total annual recruitment rate. As piglets were not modelled, the total annual recruitment rate was the rate of piglets that survived the first year, which was the product of the average local number of litters per year for yearlings/adults (*l*_*t*_), the average local litter size (*l*_*s*_) and the local probability that a piglet will not die the first year (1 − $l_{\mu }$ ). Therefore, *b*_0_ is equal to


(2.2) $$b_{0}=\frac{0.5\;l_{t}\;l_{s}\;(1-l_{\mu })}{\int_{0}^{365}\exp(-s\;\cos(\frac{\pi (t-\phi_{b})}{365}{)}^{2})dt}.$$

A factor of 0.5 is included as the model did not explicitly differentiate between wild boar sex, as in the model setting, in Eastern Europe, previous studies have found an equal ratio of adult male and female boar [48].

### 2.2. Deaths and carrying capacity

The logistic equation is a staple of ecological modelling, used to implement a population-level carrying capacity (K) ; however, it is not applicable in all contexts. The logistic equation often takes the following form:


(2.3) $$\frac{dN}{dt}=rN(1-\frac{N}{K}),$$

where N is the population size and r is the net growth rate (recruitment rate (b) − death rate (d) ). This formulation contains a density-independent term (r⁢N) and a density-dependent term $\left(r\,N^{2}/K\right)$ . This allows for a net positive rate when N<K and a net negative rate when N>K in order for the population to return to carrying capacity. When N=K , the two terms are equal, and the rate is 0. This is the first issue with equation (2.3) as there is no seasonality in the population size when the population is at carrying capacity; however, this can be remedied by allowing for seasonal variation in K .

**Figure 2.**
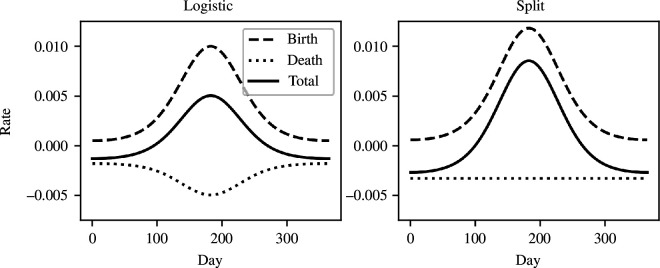
Comparison between the logistic equation and the split equations for the per pig population growth rate. The split model assumes a recruitment pulse (equation (2.1)) with *b*_0_ = 0.01, s = 3 and $\phi_{b}$ = 0, μ≈ 0.0367, σ = 0.75, θ = 1/2 and N/K = 0.5. The logistic model assumes the same recruitment pulse but a death rate ( d ) that is 2/3 the daily recruitment rate ( b⁢(t) ).

The logistic equation is a sensible formulation when r is positive, yet unwanted behaviour can occur if r is negative. If recruitment is time-dependent, for example, b⁢(t) has the form shown in equation (2.1), while d is constant and min(b(t))<d<max(b(t)) , r is positive during the birth pulse peak, but outside this time r is negative. This can cause an unrealistic timing in deaths, which occurs both when K is constant or seasonally varying. As seen in figure 2, there is a pulse of deaths in the logistic equation that mirrors the pulse in recruitment. In our model, as we did not model piglets, we would not expect an excess of deaths to occur at the same time as the recruitment pulse. Furthermore, as the maximum rate of deaths coincides with the pulse in recruitment, this formulation will incorrectly model adult wild boar deaths as studies have found that adult wild boar deaths are distributed throughout the year [44,49]. Therefore, due to the known importance of death and recruitment on disease dynamics [41], we formulated an alternative method to implement carrying capacity. We assumed that the death rate was independent of the recruitment rate and that both the recruitment and death rates contained both density-dependent and density-independent components. The new equations have the following form:


(2.4) $$f_{b}(t,N)=b(t)\left(\sigma +(1-\sigma ){\left(\frac{K}{N}\right)}^{\theta }\right),\;f_{d}(N)=\mu \left(\sigma +(1-\sigma ){\left(\frac{N}{K}\right)}^{\theta }\right),$$

where b⁢(t) is the aforementioned daily recruitment rate, σ is the modifier to determine the ratio of density-independent recruitment/deaths to total recruitment/deaths (1 is purely density-independent, whereas 0 is purely density-dependent), θ is the power of the density-dependent component (assumed to be 1/2) and μ is the daily time-independent death rate. As the average simulated model time was comparable to the average wild-boar lifetime, we assumed that K was constant.

Equation (2.4) allowed for seasonal variation in population when at carrying capacity, decoupled recruitment and deaths, and a variable split between the relative strength of density and density-independent processes. The effects of σ and its relationship to growth rate are explored in electronic supplementary material, §1. As seen in figure 2, with the *split* formulation, there is no longer the unwanted pulse of deaths mirroring the recruitment pulse; instead, the deaths are distributed throughout the year. A more complete comparison of the split formulation, logistic equation and logistic equation with a seasonally varying K , for three different ratios of N/K , is shown in electronic supplementary material, figure S2.

### 2.3. Framework structure

To analyse the effect of model structure and contact heterogeneities on ASF transmission dynamics, we developed three ASF models based on the model structure shown in figure 1. The models were

— M1—homogeneous ordinary differential equation model,
— M2—homogeneous stochastic model,
— M3—heterogeneous network-based stochastic model.

All models simulated a single isolated population of wild boar. To implement stochasticity in M2 and M3, the models were run with a Gillespie tau-leaping algorithm [50]. Models M1 and M2 were run on a homogeneous population. In M3, to include the complex wild boar social structure, the model was run on a network. The network had $N_{g}$ nodes, which represented groups of wild boar. A group could consist of multiple pigs or a solitary male boar. Edges in the network represented contact between groups, and the degree of a group ( k ) was the number of other groups a group could directly interact with. We assumed that, on average, each group had contact with six other groups ( <k>=6 ). The sensitivity of the M3 to <k> is analysed in electronic supplementary material, §9, and we found that <k>=4,8,10 neither offered a better quality fit nor appreciably changed dynamics.

**Figure 3.**
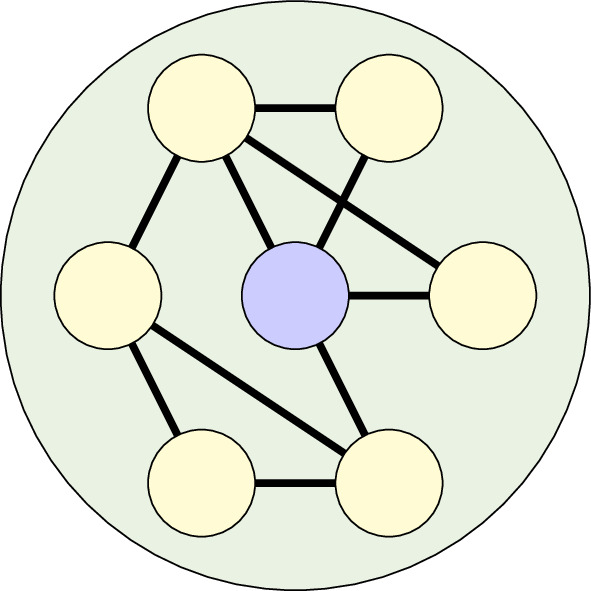
Network model population structure. Sow groups are yellow, while solitary boar *groups* are blue. Inter-group connections are shown by solid black lines.

Previous wild boar studies have found that male boars have larger home ranges than females [51,52]. Therefore, we assumed that the larger home ranges of male boar would result in an increased average number of contacts. To include this in the model, the nodes with the highest degree were assigned to be a solitary male boar, while the remaining nodes were sow groups (of size $N_{s}$ ). An indicative population structure of M3 is given in figure 3.

Network type is known to influence epidemic dynamics [53]; however, as the true network is unknown, we used several algorithms to generate different idealized networks with varying characteristics that cover a range of attributes to investigate the effect of network type. The following three networks were tested:

— Erdős–Rényi random (RN) network [54],
— Barabási–Albert scale-free (SF) network [55],
— Watts–Strogatz small-world (SW) random network [56].

In the RN network models, the degree distribution of each group follows a Poisson distribution. Both clustering and average path length were low. The SW network in this study was run with a rewiring probability of 0.2, which allowed the network to have high clustering and low average path length. In SF networks, the degree distribution follows a power-law ( $P\,\left(k\right)\propto k^{-3}$ ), such that <k> is finite, but the variance is infinite. Therefore, most groups had low connectivity, but some groups (solitary boars) were highly connected and could act as key hubs (nodes with high betweenness centrality) in the network. The average clustering in the SF networks generated for this study was low.

### 2.4. Transmission

There were two ASF transmission pathways in the framework: infection from live pigs or infection from infectious carcasses of pigs. In the homogeneous models, M1 and M2, both pathways were governed by a single transmission rate, $\beta_{h}$ . In M3, an infection could come from within a pig’s group or from a connected group; hence, there were two rates—intra-group transmission ( $\beta_{i}$ ) and inter-group transmission ( $\beta_{o}$ ). To determine the role of carcasses in dynamics, transmission from carcasses was scaled by ω , the relative infectivity of carcasses to live pigs, which was a fitted parameter.

As the decay of infective carcasses is highly dependent on temperature [21], we modelled decay times with a sinusoidal function to account for the seasonal change in temperature in the study location. Carcass decay had the following form:


(2.5) $$\lambda (t)=\lambda_{0}+\lambda_{1}\;\cos\left(\frac{2\pi (t+\phi_{\lambda })}{365}\right),$$

where $1/\lambda_{0}$ is the carcass decay period, $1/\lambda_{1}$ is the magnitude of the seasonal change in carcass decay period, and $\phi_{\lambda }$ is the carcass decay timing offset.

**Figure 4.**
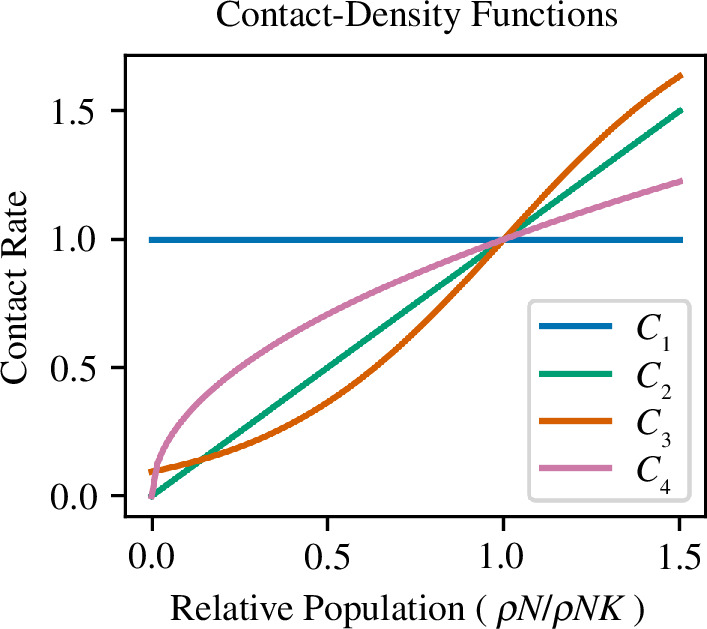
Plots of the four contact-density functions tested in the models. The frequency-based contact function (*C*_1_) is in blue, the density-based contact function (*C*_2_) is in green, the sigmoid contact function (*C*_3_) is in orange and the power-law contact function (*C*_4_) is in pink.

In wild boar, higher densities probably lead to high contact rates [57]; however, the exact contact-density function is not known. A positive relationship between density and disease spread has been noted at the population level in both classical swine fever studies [58–60] and ASF studies [24,61,62]. Furthermore, power functions have been shown to offer a realistic scaling of transmission rate with density in many ecological settings [63–65]. Therefore, similar to Borremans *et al.* [63], we tested a number of contact-density functions that scaled with density. We set $\beta_{h/o}=C\,\left(\rho_{N}\right)\,p$ , where $C\,\left(\rho_{N}\right)$ is the contact rate, a dimensionless scaling factor that is a function of population-level density ( $\rho_{N}$ ), and p is the transmission rate. We tested four formulations of $C\,\left(\rho_{N}\right)$ for $\beta_{h}$ and $\beta_{o}$ ; these relationships are given in table 1 and plots of how each formulation scales with density are shown in figure 4. Importantly, populations in all models were assumed to occupy a constant area. This area was calculated at the start of each simulation given the population density at carrying capacity $\left(\rho_{K}\right)$ and K . At each time step, this area is used to calculate boar density ( $\rho_{N}$ ).

**Table 1.** The four contact-density functions tested in the models. Here, $C\,\left(\rho_{N}\right)$ is the contact-density function, $\rho_{N}$ is the wild boar density (total pigs per unit area) and $\rho_{K}$ is the wild boar density at K .

function	transmission type	description
*C*_1_	frequency based	$C\,\left(\rho_{N}\right)=1$
*C*_2_	density based	$C\,\left(\rho_{N}\right)=\rho_{N}/\rho_{K}$
*C*_3_	sigmoid	$C\,\left(\rho_{N}\right)=\tanh\left(1.5\,\rho_{N}/\rho_{K}-1.5\right)+1$
*C*_4_	power-law	$C\,\left(\rho_{N}\right)={\left(\rho_{N}/\rho_{K}\right)}^{1/2}$

In M3, unlike inter-group transmission, intra-group transmission was density-independent. Bouma *et al.* [66] found that intra-group pseudo-rabies transmission in pigs, on the group scale, was independent of population density. Therefore, intra-group transmission was assumed to be frequency-based and the force of infection for a group i in M3 has the form


(2.6) $$\lambda (I,C,N)=p_{i}\frac{I_{i}+\omega C_{i}}{N_{i}+C_{i}}+\sum_{j=1}^{n}C_{ij}\left(\rho_{N}\right)p_{o}\frac{I_{j}+\omega C_{j}}{N_{i}+C_{i}+N_{j}+C_{j}},$$

where the first term is the density-independent intra-group transmission, and the second term quantifies inter-group transmission. $C_{i\,j}\,\left(\rho_{N}\right)$ is the contact-density function between two groups i and j , which was equal to zero if the two groups were not connected by the network or if i=j . Similarly, for M1 and M2, the force of infection had the following form:


(2.7) $$\lambda (I,C,N)=C(\rho_{N})p_{h}\frac{I+\omega C}{N+C}.$$

The stochastic models are parametrized by 11 distinct transitions, which rely on the recruitment, death and transmission processes outlined above, along with other disease progression processes. The transitions for M2 are outlined in electronic supplementary material, §4, all transitions for M3 are outlined in electronic supplementary material, §5, and the underlying ODE equations of M1 are outlined in electronic supplementary material, §3.

## 3. Fitting models to recent outbreak data

### 3.1. Parameters and model fitting

All models relied on two sets of parameters: transmission parameters which were assumed the same across populations $\left\{p_{h},p_{o},p_{i},\omega ,\sigma ,\theta ,\zeta ,\gamma ,\kappa ,\nu \right\}$ , and population and location-specific parameters $\left\{K,\rho_{K},<k>,N_{g},N_{s},\varkappa ,l_{y},l_{s},l_{\mu },\mu ,\phi_{b},\lambda_{0},\lambda_{1},\phi_{\lambda }\right\}$ . Transmission parameters— $p_{h},p_{o},p_{i},$ and ω , were estimated by fitting the candidate models to epidemiological criteria derived from the recent ASF outbreaks in the Baltic States. Transmission parameters are given in table 2, while population parameters are given in table 3.

**Table 2.** Transmission-based model parameters.

parameter	description	value	source
*p*_*h*_	homogeneous transmission rate (M1 and M2)	fitted	**–**
*p*_*o*_	inter-group transmission rate (M3)	fitted	**–**
*p*_*i*_	intra-group transmission rate (M3)	fitted	**–**
ω	relative infectiousness of carcasses to live pigs	fitted	**–**
σ	proportion of density-independent recruitment and deaths to total recruitment and deaths	0.75	[48,67]
θ	power coefficient of density-dependent recruitment and deaths	0.5	assumption
1/ζ	latent period	6 days	[68–70]
1/γ	infectious period	8 days	[69–71]
1/κ	waning immunity period	180 days^a^	
ν	lethality	0.95	[1,74]

^a^ Immunity duration is a current knowledge gap and requires further investigation [72]. Sereda *et al.* [73] found that immunization with attenuated ASFV strains offered at least four months protection from virulent ASFV strains.

**Table 3.** Population model parameters selected to reflect the Baltic States.

parameter	description	value	source
K	carrying capacity of modelled region	5000 pigs	assumption
$\rho_{K}$	initial wild boar density	2.8 pigs km^-2^	[48]
<k> ^a^	average number of inter-group connections	6 connections	assumption
$N_{g}$ ^a^	number of groups	1000 groups	assumption
$N_{s}$ ^a^	sow group size	6 pigs	[48,75,76]
χ ^a^	ratio of solitary boar groups to total groups	0.2	[75]
*l*_*y*_	number of yearly litters	0.9 litters	[77,78]
*l*_*s*_	litter size	5.8 pigs	[47,48,78,79]
$l_{\mu }$	first-year mortality	0.5	[48,49,78]
μ	death rate at K	0.0036 day^−1^	$0.5l_{y}l_{s}(1-l_{\mu })/365$
s	birth pulse width	3	[47]
$\phi_{b}$	birth pulse offset	75 days	[47]
$1/\lambda_{0}$	base carcass decay period	60 days	local climate [21]
$1/\lambda_{1}$	seasonal change in carcass decay period	30 days	local climate
$\phi_{\lambda }$	carcass decay offset	0 days	local climate

^a^ Parameters only used in heterogeneous network model.

The models were fitted using an approximate Bayesian computation with a sequential Monte Carlo algorithm (ABC-SMC). Each model had an initial population of 5000 wild boar, which was the assumed carrying capacity of the modelled region. All models assumed that ASFV was introduced during the summer, as wild boar tend to have larger home ranges during this season [80]. In M1 and M2, ASF was initialized with 1% prevalence, while in M3 ASF was seeded in five connected groups. This high number was chosen to minimize the occurrence of minor outbreaks in the stochastic models [81]. In M1 and M2, which assumed homogeneous populations, the fitted parameters were *p*_*h*_ and ω . In M3, the fitted parameters were *p*_*i*_, *p*_*o*_ and ω . All parameters had semi-informative uniform priors: $p_{h}∼$ Unif(0,1.0), $p_{i}∼$ Unif(0,1.0), $p_{o}∼$ Unif(0,0.25), ω∼ Unif(0,1.0). All fitting simulations were run for 6 years. As there was limited high-resolution temporal data on ASF incidence in wild boar populations in the study location, model fit was judged against summary statistics. The following three summary statistics were derived from characteristics of the ASF outbreaks in the Baltic States, where ASF has continued to circulate in wild boar populations since 2014 [82]:

— S1—The prevalence of ASFV several years post-introduction is 1.5% (95% CI [1%, 2%]) [82,83].
— S2—75% decline in wild boar density during the epidemic (95% CI [65%, 85%]) [4,84,85].
— S3—Infection peak occurs 180 days after the first detected case (95% CI [120 days, 240 days]) [4]. As there was no surveillance in the model, we assumed the first detected case would occur at 5% prevalence.

To assess the effect of contact-density functions, every model was fitted with the four contact functions (table 1). Furthermore, to analyse the effect of network structure, M3 was also fitted with the three network types (RN, SF and SW).

### 3.2. Homogeneous model fitting results

In both M1 (ODE) and M2 (homogeneous stochastic) models, the posterior distributions of the fitted parameters for each contact-function sub-model showed largely the same behaviour. Properties of the posteriors for *p*_*h*_ and ω are given in electronic supplementary material, table S3, and plots of the M1 and M2 posterior distributions are given in electronic supplementary material, figures S6 and S7. To assess the quality of fit of M1 and M2, for each contact-density formulation, we simulated both M1 and M2 using draws from the fitted posterior distributions. Each sub-model was run 10 000 times, and simulations ran for 6 years. Summary statistics for each run were computed and compared with the observed summary statistics.

**Figure 5.**
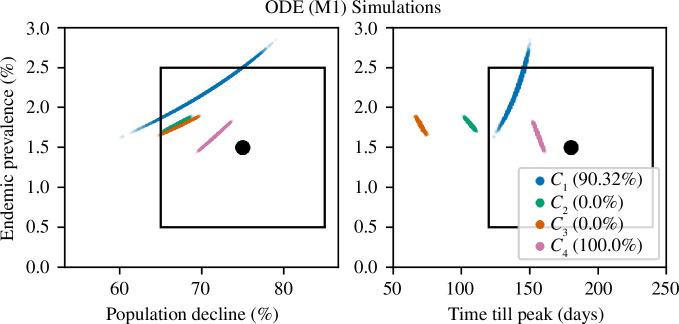
Comparison between the three simulated summary statistics and the observed summary statistics for ODE model (M1). The plot on the left compares statistics S1 and S2, while the right plot compares S1 and S3. The point estimates of the observed summary statistics are represented by the black dot and the associated 95% confidence regions by the black rectangles. Results from the frequency-based contact function (*C*_1_) are in blue, from the density-based contact function (*C*_2_) in green, from the sigmoid contact function (*C*_3_) in orange and from the power-law contact function (*C*_4_) in pink. All models were simulated 10 000 times. In brackets in the legend are the proportion of simulations where all three simulated summary statistics were within the 95% CI of all observed summary statistics.

The results for the M1 simulations are given in figure 5. The best fitting contact-density function was the power-law relationship $\left(C_{4}\right)$ , where 100% of the simulated summary statistics were within the 95% CIs of all observed summary statistics. The next most effective contact-density function was frequency based (*C*_1_), where 90% of simulated summary statistics were within the 95% CI of all observed summary statistics. No simulated summary statistics for the density-based transmission (*C*_2_) and the sigmoid transmission (*C*_3_) were within the 95% CI of all observed summary statistics. Long-term prevalence and population decline were within the 95% CI; however, ASF spread too quickly, and the peak in infections occurred prematurely (on average in 106 days for *C*_2_ and in 69.6 days for *C*_3_).

**Figure 6.**
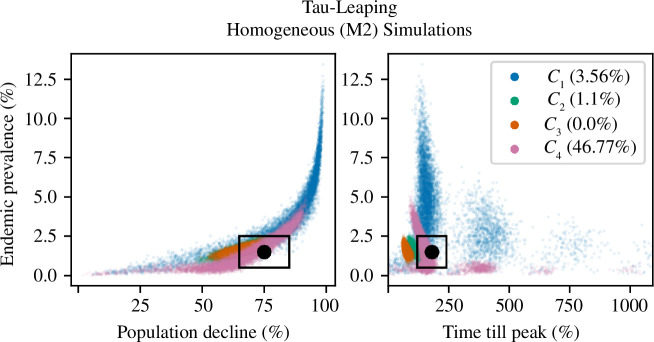
Comparison between the three simulated summary statistics and the observed summary statistics for the stochastic homogeneous model (M2). The plot on the left compares summary statistics S1 and S2, while the right plot compares S1 and S3. The observed summary statistics are represented by the black dot and the associated 95% CI by the black rectangle. Results from the frequency-based contact function (*C*_1_) are in blue, the density-based contact function (*C*_2_) in green, the sigmoid contact function (*C*_3_) in orange and the power-law contact function (*C*_4_) in pink. Each contact-density formulation model was simulated 10 000 times. In brackets in the legend are the proportion of simulations where all three simulated summary statistics were within the 95% CI of all observed summary statistics.

While similar to the M1 results, the stochastic nature of M2 caused a greater spread in simulated summary statistics (figure 6). Contact function *C*_4_ again produced the most accurate model, with approximately 47% of simulated summary statistics lying within the 95% CIs of all observed statistics, and ASFV persisted in over 97% of simulations after 6 years. All other contact functions produced a poor fit and were largely unable to reproduce the observed summary statistics. Only 4% of simulated *C*_1_ summary statistics were within all 95% CIs, and after 6 years, ASFV was present in 70% of simulations. Furthermore, long-term prevalence was often too high; a third of simulations that had ASFV present at the end of the simulation period had an average ASF prevalence greater than 5%.

**Figure 7.**
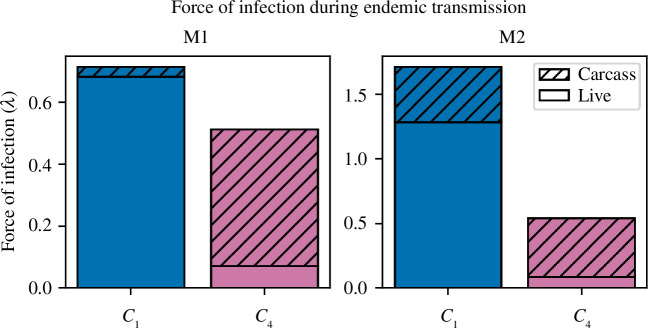
The yearly force of infection ( 𝝀 ) from carcasses and live pigs, computed for the ODE model (M1) and the homogeneous tau-leaping model (M2), with both frequency-dependent transmission (blue) and the power-law contact-density function (pink). The force of infection was calculated between years 3 and 6 in simulations where ASF was present at the end of the simulation period.

In the homogeneous models (M1), the two most accurate contact-density functions, *C*_1_ and *C*_4_, predicted different dominant transmission pathways. To analyse the various routes of ASF transmission, the aforementioned simulations were analysed to calculate the force of infection from live pigs ( ${𝝀}_{𝒍}$ ) and from pig carcasses ( ${𝝀}_{𝒄}$ ). The force of infection was only calculated between years 3 and 6 in simulations where ASF was present at the end of the simulation period. Results are given in figure 7, and results with confidence intervals are given in electronic supplementary material, table S5. In M1, the *C*_1_ sub-model predicted ${𝝀}_{𝒍}\approx 20\,{𝝀}_{𝒄}$ , while the converse was true in the *C*_4_ sub-model, where ${𝝀}_{𝒍}\approx \frac{1}{6}\,{𝝀}_{𝒄}$ . Therefore, transmission in the *C*_4_ sub-model was primarily driven by carcasses, while in the *C*_1_ sub-model, transmission was dominated by live pigs, and there was comparatively little carcass-based transmission. Furthermore, the total force of infection in the *C*_1_ sub-model was 36% larger than in the *C*_4_ sub-model.

In the stochastic homogeneous model (M2), there was a similar, albeit lesser, dichotomy in transmission predicted by *C*_1_ and *C*_4_. The *C*_1_ sub-model predicted ${𝝀}_{𝒍}\approx 3\,{𝝀}_{𝒄}$ , while in the *C*_4_ sub-model, ${𝝀}_{𝒍}\approx \frac{1}{5}\,{𝝀}_{𝒄}$ . The total force of infection of the *C*_1_ sub-model was 220% larger than in the *C*_4_ sub-model and was 140% greater than in the M1 *C*_1_ sub-model. This high force of infection may stem from the inability of the M2 *C*_1_ sub-model to accurately reproduce the observations, as in a number of simulations, the long-term prevalence was too high, which would have caused an overestimation of the force of infection. The total force of infection calculated from the most accurate models (M1 *C*_1_, M1 *C*_4_ and M2 *C*_4_ sub-models) was comparable to that calculated by Loi *et al.* [86], who estimated the force of infection of ASF in wild boar in northern Sardinia. For completeness, the basic reproduction number (*R*_0_) and the effective reproduction number ( $R_{e\,f\,f}$ ) for M1 and M2 were calculated. Results are presented in electronic supplementary material, §8.

The fitting procedure outlined above was repeated for a logistic-equation-based ODE (LM1) and homogeneous tau-leaping model (LM2) to quantify the effect of using the *split* equation over the logistic equation on ASF dynamics. For LM1, the *C*_1_ and *C*_2_ functions could accurately reproduce the observed summary statistics; however, in LM2, no contact-density sub-model was able to adequately replicate the summary statistics. The results of the logistic-based simulations are given in electronic supplementary material, §1.2.

### 3.3. Heterogeneous model fitting results

For the heterogeneous tau-leaping model (M3), an ensemble of six sub-models were fitted (contact functions *C*_1_ and *C*_4_ run on each of the three network types). *C*_2_ and *C*_3_ sub-models were excluded due to their poor fit in M1 and M2. The properties of the posterior distributions of intra-group transmission (*p*_*i*_), inter-group transmission (*p*_*o*_) and relative infectivity of carcasses ( ω ) are given in electronic supplementary material, table S4, while plots of the posterior distributions are given in electronic supplementary material, figure S9.

**Figure 8.**
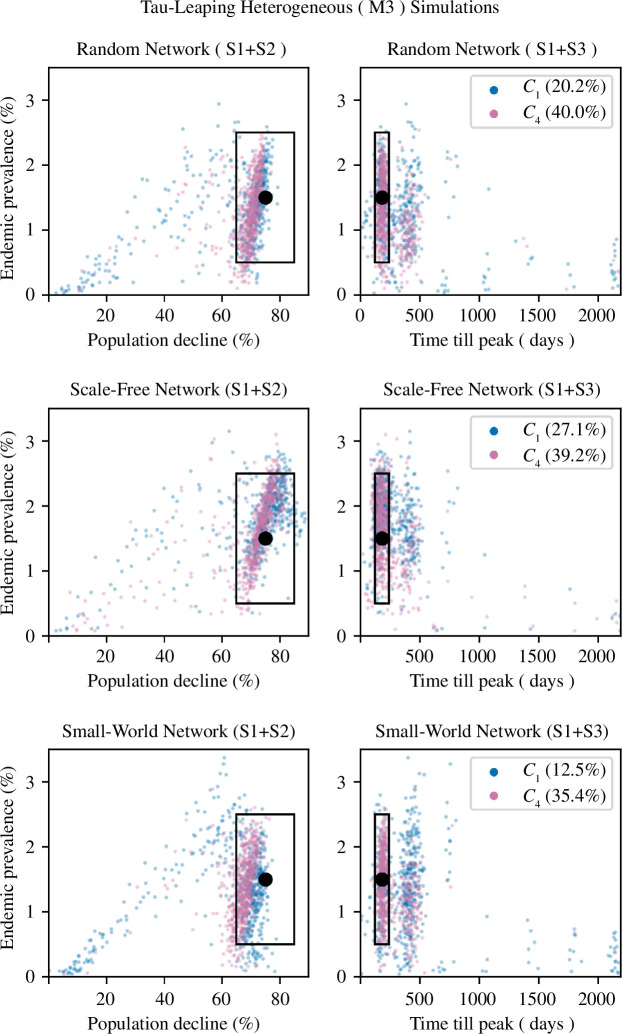
Comparison between the three simulated summary statistics and the observed summary statistics for the tau-leaping heterogeneous model (M3). Each row is the M3 model run on a different network. The left plots compare summary statistics S1 and S2, while the right plots compare S1 and S3. The observed summary statistics are represented by the black dot and the associated 95% CI by the black rectangle. Results from the frequency-based contact function (*C*_1_) are in blue and from the power-law contact function (*C*_4_) are in pink. Due to computational limitations, each model was simulated 1000 times. In brackets in the legend is the percentage of simulations where all three simulated summary statistics lie within the 95% CI of all observed summary statistics.

To assess the goodness of fit of the six M3 models, we again compared summary statistics from simulations using their fitted posteriors with the observed summary statistics. Each model was simulated 1000 times, and the results are given in figure 8. Unsurprisingly, the increased heterogeneity in M3 caused greater variability in summary statistics. Across all networks, the *C*_4_ sub-models were better able to reproduce the observed summary statics than *C*_1_ sub-models. The *C*_4_ sub-models could better model continued persistence; after 6 years, ASFV was present in 51.5% of *C*_1_ simulations and 62% of *C*_4_ simulations. There were also notable differences between networks. SW networks, for both contact functions, had the lowest proportion of simulated summary statistics within the 95% CIs of all observed summary statistics. On both SF and RN networks, *C*_4_ simulations had similar levels of fit, while a greater proportion of SF *C*_1_ simulations were within the observed summary statistic confidence intervals.

Compared with the homogeneous stochastic model (M2), depending on the contact function, the M3 model offered comparable or better levels of fit. The M3 *C*_1_ sub-models had an up to seven times greater proportion of simulations within all observed confidence intervals than the M2 *C*_1_ sub-model. In contrast, the M2 *C*_4_ sub-model had, on average, a 1.22 times greater proportion of simulations within the observed confidence intervals than the M3 *C*_4_ sub-models.

**Figure 9.**
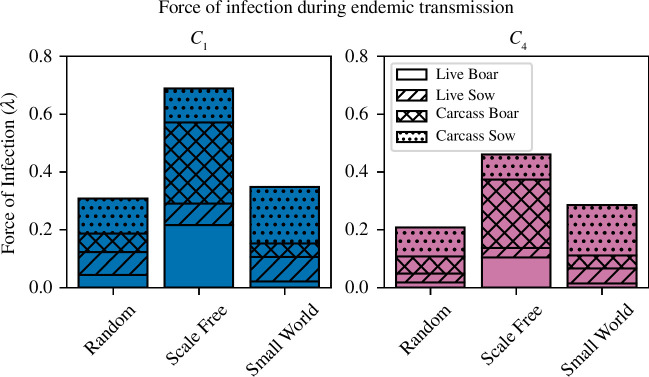
The force of infection ( 𝝀 ) calculated for the tau-leaping heterogeneous model (M3). On the left, in blue, are sub-models that assume a frequency-based contact-density function and the right plot, in pink, are sub-models that assume a power-law contact-density function. The unhatched bars are the force of infection from live boars, the diagonal hatch bars are the force of infection from live sow, the cross-hatch bars are the force of infection from boar carcasses and the dot-hatch bars are the force of infection from sow carcasses. The force of infection was calculated between years 3 and 6 in simulations where ASF was present at the end of the 6 year simulation period.

To analyse the different routes of ASF transmission in M3, the six sub-models were again run from their fitted posteriors, simulated 1000 times, and the force of infection was calculated between years 3 and 6 in simulations where ASF was present at the end of the simulation period. We decomposed the force of infection into components from living pigs and pig carcasses, and from solitary boar $\left({𝝀}_{𝒃}\right)$ or sow groups $\left({𝝀}_{𝒔}\right)$ . The results for inter-group transmission are given in figure 9, and results with confidence intervals are given in electronic supplementary material, table S6. As the primary focus of the heterogeneous model was to investigate how ASF spreads through the network and because the force of infection has limited utility within groups (due to small sow group size and solitary boar groups), the force of infection was only computed for inter-group transmission. Consequently, the magnitudes of the calculated forces are not directly comparable with those from the homogeneous models (M1 and M2).

Infected carcasses were the dominant pathway of transmission for every network type and both contact-density functions. Similar to what was observed for M1 and M2, the total force of infection in the frequency-based (*C*_1_) sub-models was greater than that for power-law-based (*C*_4_) sub-models. Network structure influenced the force of infection, particularly the relative importance of sows or boars. Of the tested networks, SW models had the lowest relative transmission from solitary boars as ${𝝀}_{𝒃}\approx 0.25\,{𝝀}_{𝒔}$ . In RN models, sows were still the primary drivers of infection; however, boars had a greater influence on ASF spread as ${𝝀}_{𝒃}\approx 0.56\,{𝝀}_{𝒔}$ . Conversely, the SF models were dominated by boar-based transmission as ${𝝀}_{𝒃}\approx 2.69\,{𝝀}_{𝒔}$ . Therefore, in the SF models, although solitary boars only comprised 4% of the population, they were responsible for approximately 70% of inter-group transmission. This large influence of boars is explained by the SF network structure; the highly connected boars ( k≫<k> ) act as hubs in the group network and drive inter-group transmission.

## 4. Intervention

**Figure 10.**
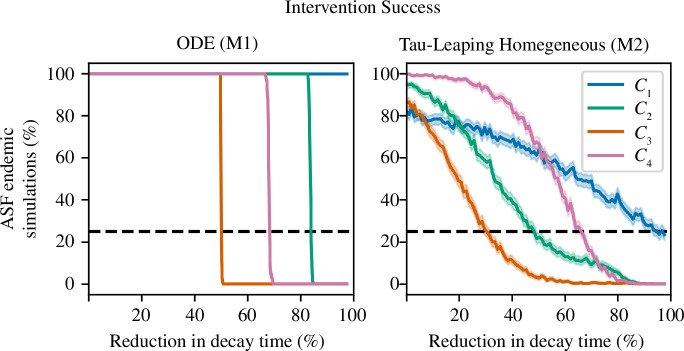
The effectiveness of a carcass removal intervention in both the ODE model (M1) and the tau-leaping homogeneous model (M2). Each model investigates the impact of different assumed contact-density functions: frequency-based (*C*_1_) in blue, density-based (*C*_2_) in green, sigmoid-based (*C*_3_) in orange and power-law-based (*C*_4_) in pink. 95% credible intervals are given by the lighter-shaded region.

To explore the impact of model choice on potential programmatic outcomes, we simulated an idealized ASF intervention. Specifically, we analysed the efficacy of removing wild boar carcasses to prevent long-term ASFV transmission, as Guinat *et al.* [87] and Gervasi & Guberti [88] found that carcass removal can be a highly effective, albeit potentially impractical, intervention. Interventions began 90 days after ASFV was seeded in the population and continued for 3 years. Interventions were modelled by reducing the carcass decay period ( 1/λ ) by a multiplicative factor ( α ), where α∈[0,0.975] . While a 97.5% reduction in decay time and the removal of all pig carcasses is unachievable in practice, these interventions were considered for illustrative purposes. The effect of the intervention was tested for all combinations of model types (M1–M3), contact-density functions (*C*_1_ *–C*_4_), and networks (RN, SF and SW). For the two homogeneous models, M1 and M2, each magnitude of α tested was simulated 1000 times. As in §3, $p_{h},p_{i},p_{o}$ and ω were simulated from their fitted posteriors. The results for M1 and M2 are given in figure 10.

Results from M1 highlight potential limitations with simple homogeneous ODE models. We found that in the *C*_1_ sub-model, carcass removal did not reduce the number of simulations with ASF transmission after 3 years; all simulations had 100% persistence after the intervention period. The lack of intervention success occurs because carcass-based transmission in *C*_1_ is negligible. The remaining three ODE models exhibited a sudden transition, from ASFV persistence in 100% of simulations to ASFV persisting in no simulations. This bifurcation required an approximate reduction in decay period of 50% for *C*_3_ sub-models, 68% for *C*_4_ sub-models and 84% for *C*_2_ sub-models. The abrupt shift between the two states is unrealistic and may give a false prediction of potential programmatic success.

M2, which included stochasticity, did not display the sharp transition between endemicity and extinction that occurred in M1. Instead, compared with M1, all sub-models were characterized by a gradual increase in the number of simulations with ASFV extinction with an increase in the magnitude of α . ASFV extinction in 75% of simulations required an average reduction in the decay period of 97% for *C*_1_ sub-models, 66% for *C*_4_ sub-models, 49% for *C*_2_ sub-models and 30% for *C*_3_ sub-models. Unlike in M1, the *C*_1_ sub-model did respond to the intervention; however, again, due to the negligible levels of carcass-based transmission, the required intervention was large and would probably not be practically achievable. The addition of stochasticity changed the ordering of the intervention efficacy of sub-models. Importantly, the simple addition of stochasticity removed the sudden bifurcation present in M1 and produced more realistic responses to the intervention.

**Figure 11.**
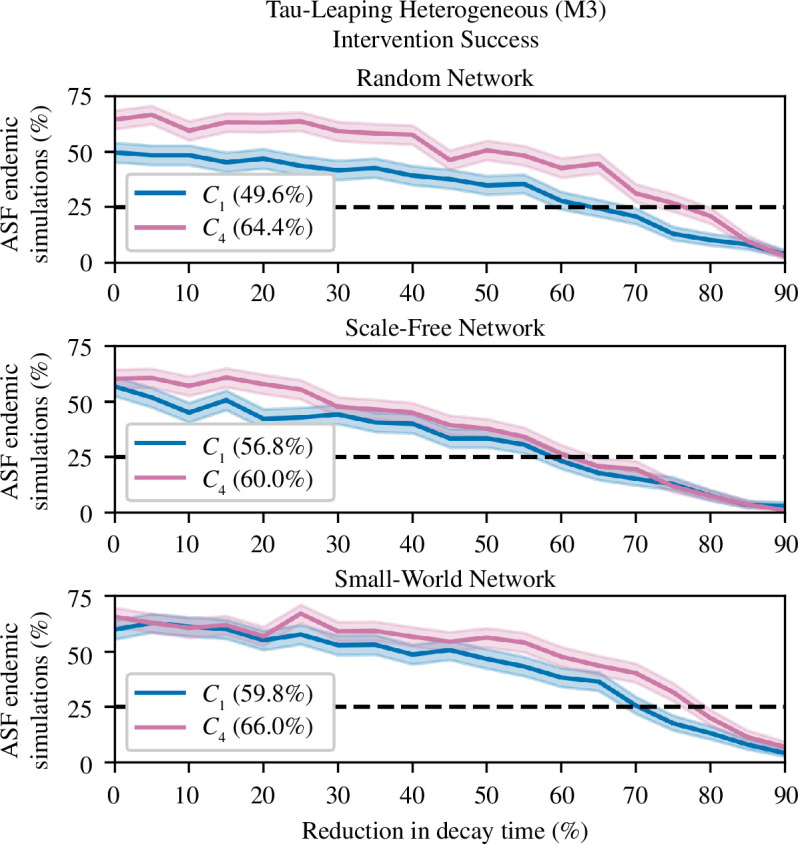
The effectiveness of a carcass removal intervention in the tau-leaping heterogeneous model (M3). Each row is the model run on a different network structure. In each plot, the model investigates the impact of different assumed contact-density functions—frequency-based (*C*_1_) in blue and power-law-based (*C*_4_) in pink. In brackets in the legend is the proportion of simulation where ASF is present after 3 years with no intervention.

In M3, both network and contact-density function types were found to influence intervention success. For the M3 interventions, each magnitude of α was simulated 500 times. As with the previous analysis of M3, only *C*_1_ and *C*_4_ sub-models were included. All interventions were simulated for 3 years and began 90 days after ASFV was seeded in the population. For each network, after normalizing for intervention-free fade-out, *C*_1_ sub-models required comparable or lesser intensity of intervention to achieve elimination than required by the *C*_4_ sub-models (figure 11). The increased effectiveness of the intervention in *C*_1_ M3 models, when compared with the *C*_1_ homogeneous models, occurs as the *C*_1_ M3 models have a greater relative proportion of transmission from carcasses.

Network choice influenced the likelihood of intervention success. We found that the SF network, on average, required the lowest intensity of intervention to reach the 75% elimination benchmark, while SW and RN models required similar intensities of intervention. The better performance of the intervention on the SF network was unsurprising due to the aforementioned SF network structure and the key role solitary boar play in transmission. If a highly connected boar becomes infected, dies, and their carcasses are promptly removed via the intervention, this can quickly remove a highly connected node. This can disconnect regions of the network, limit spread and cause fade-out. Similar behaviour has been noted in previous SF studies [53,89].

## 5. Conclusion

Previous wild boar-focused ASF modelling studies have primarily relied on ODE or IBM. The former often omits wild boar social structure, while the latter does include social structure. However, limited research has been done on the effect of inter-group contact structure on ASF transmission in wild boar populations. A better understanding of contact structure is crucial as wild boar populations exhibit significant contact heterogeneity, which can affect transmission and potential interventions [90]. Furthermore, IBM, while often providing the most accurate abstraction of a system and an intuitive application of the spatial components of the transmission, can be computationally expensive and require a significant number of parameters, which may be challenging to measure due to the knowledge gaps around ASF transmission in wild boar [30], social structures [37] and inter-group and carcass contact rates [91].

We investigated the impact of various contact-density functions, stochasticity and the addition of contact networks on transmission dynamics. Across all models, the power-law $\left(C_{4}\right)$ contact-density function was best able to replicate the observed summary statistics from previous outbreaks in the Baltic States. The nonlinear dependence of contact rate on density, where contact rates saturate at higher densities, has been observed in previous human and wildlife diseases [64,65]. Furthermore, the addition of contact networks reduced the differences between the contact-density functions. All tested networks had approximately comparable levels of fit; however, they predicted significantly different forces of infections from solitary boars and sow groups. In SF network simulations, solitary male boars had, on average, significantly more contacts than sow groups due to their larger home ranges and the power-law degree distribution. Due to the network’s high betweenness centrality, solitary boar groups accounted for a majority of transmission. This very high connectivity of certain highly mobile solitary boar groups is probably unrealistic, as Podgórski & Śmietanka [92] found that wild boar movements were poor predictors of ASF dynamics as increased movement may not correlate to increased contacts. The remaining two tested contact networks found that less-connected multi-pig groups were the key drivers of transmission. SW and RN networks displayed approximately the same long-term response to interventions, despite the different levels of network clustering. This was unsurprising as clustering plays a limited role in determining epidemic probability and size [93].

We found that carcass-mediated transmission was the key driver to long-term ASF persistence. In all *C*_4_ models, the force of infection from carcasses was greater than the force of infection from live boars ( $\lambda_{c}>\lambda_{l}$ ). This aligns with Pepin *et al.* [25], who found that in Eastern Poland, a majority of transmission events were carcass-based, and Gervasi and Guberti [26] who found that carcass-based transmission events are dominant during the endemic phase of transmission. Additionally, increased model heterogeneity led to a decrease in the relative importance of carcass-based transmission. However, the strong influence of carcasses on dynamics highlights the potential utility of carcass-based interventions to control ASF spread and persistence.

There are limitations to the modelling presented in this study. Firstly, the populations were modelled as isolated systems of 5000 pigs. This is unrealistic as the populations in the Baltic States are not isolated, as evidenced by multiple wild boar-mediated ASF incursions [94]. Furthermore, transmission in these wild boar populations has been aided by human activity [19]. The model also excludes piggeries, which could be a worthwhile future extension of the work, as the interface between wild and domestic pigs is an important factor of ASF outbreaks [30].

The model of recruitment may have affected transmission and population dynamics. Piglets were not modelled due to often high mortality rates; their addition would further increase the recruitment of susceptibles in the model during birthing periods, which could influence ASF dynamics and allow for more spread around these birthing periods. Recruitment of adult pigs was also instantaneous and not proportional to the population of the previous year. This could affect both population and ASF transmission dynamics. Furthermore, yearlings and adult pigs were in the same class. Yearlings and adult pigs have similar but different characteristics and behaviours, for example, different litter sizes and mortality rates [48].

There is a high degree of uncertainty surrounding a number of key processes in wild boar populations, and ASF transmission. The summary statistics used to fit the model may be affected by under- or over-reporting, and model fit could be improved by the inclusion of more detailed spatio-temporal data. Although carcass decay and recruitment had a strong time dependence, the transmission rate ( β ) did not. Wild boar contact rates vary by season [44]; therefore, there should be a degree of seasonality to β . Time dependence in transmission has been shown to alter potential outbreak sizes, induce complex dynamics in endemic scenarios, and reduce disease persistence [95]. All networks were static in both nodes and connections, which is probably unrealistic over the 6 year simulation period. Another limitation of the network, is that after a boar dies, it remains connected to all their previously connected groups until their carcass decays. Boars were chosen to be the highly connected nodes on the basis that they have the largest home range, meaning a boar would travel and contact many other boar and sow groups. Once a boar dies, this travel cannot occur, but in our model, the boar remains connected to the same number of other groups. Therefore, the model may overestimate the force of infection from boar carcasses, especially within the SF network. Issues around abstracting the wild boar contact systems face both network-based models and IBM, as both require a number of assumptions and parameters to model an uncertain system. The advantage of the network-based approach is limiting the complexity to the transmission side of the model while streamlining the model of wild boar ecology.

ASF outbreak dynamics are highly dependent on model specification. Specifically, we found several factors necessary to account for when modelling ASF transmission. The first is that models should include stochasticity. As seen in the results of the M1 model, the deterministic nature gave a potentially unrealistic level of fit, which, in turn, could cause false confidence in the model’s predictive accuracy. This is compounded by the improbable bifurcation response to interventions, which further highlights the model’s limitations. Secondly, as wild boar are highly social, proper consideration should be given to modelling transmission. The relationship between boar density and transmission rate must be accounted for as it is probably neither purely density-based nor frequency-based. It may be best represented by sub-linear functions that saturate at higher densities. The form of the contact-density function may affect the dominant transmission pathways, which can affect potential responses. Boar social structure can be naturally expanded to include group structure on a network and, if run on a network, network choice impacts the relative importance of sow groups, boar groups, live and carcass-based transmission. Compared with homogeneous stochastic models, explicit group structure affects the variability of long-term prevalence, dominant sources of infection, and intervention efficacy. Furthermore, in wildlife populations, network structure greatly influences the intensity of outbreaks for highly transmissible diseases [96], such as ASF. Finally, correctly modelling carcass-based transmission is essential. This study and the recent ASF literature show that wild boar carcasses play a key role in long-term ASF transmission. Therefore, to accurately model ASF in different geographic locations, it is crucial to understand the period of viability of ASFV in carcasses in different climatic conditions, e.g. humidity and temperature. Overall, this study provides insights into transmission dynamics and model complexity, which are essential for accurately modelling ASF transmission in wild boar populations.

## Data Availability

Data and relevant code for this research work are stored in [97]. Electronic supplementary material is available online [98].
